# 11.J. Workshop: Enabling participation of vulnerable groups in primary health and long-term care

**DOI:** 10.1093/eurpub/ckac129.717

**Published:** 2022-10-25

**Authors:** Organised by: Bielefeld University (Germany), Chair persons: Kerstin Haemel (Germany), Anja Dieterich (Germany)

## Abstract

Enabling individual and collective participation or vulnerable people in health and social care is fundamental to achieving equitable, people-centered care. However, for vulnerable groups and clients, unfolding opportunities for participation in their care is closely related with experiences of empowerment and disempowerment. The workshop focuses on primary health and long-term care, as those are the pillars of resilient health care systems able to meet the complex care needs of vulnerable people. From a public health perspective, the facets of participation range from providing access to comprehensive services for people in need, to developing a shared understanding between clients and health professionals regarding clients’ everyday and care preferences, to sharing decision-making on health and care matters. In this workshop, we will discuss how helpful public health interventions between individual, professional and systemic-structural approaches should or could be designed, to (better) enable participation of vulnerable groups in primary and long-term care. The workshop presentations draw on empirical research with members of vulnerable groups, health professionals and key informants in care policy and practice conducted in Brazil, Germany, India, and Spain. By focusing on different primary and long-term care settings as well as vulnerable groups (undocumented persons, older people in need of care, people living with chronic illnesses) we will address and discuss the following questions:

• What ideas can we find to strengthen participation of vulnerable groups in primary and long-term care settings?

• Which opportunities and barriers are involved?

• How can individual, professional, and structural approaches ‘fit together’ to enable greater participation of vulnerable groups in their (self-)care?

**Key messages:**

• Practitioners’ efforts to enable participation have to be accompanied by qualification- and governance-oriented public health approaches to achieve patient oriented health and social care systems.

• Present barriers and opportunities for vulnerable groups are instructive indicators for broader challenges towards comprehensive people-centered care.

